# P-1843. Real-world experience and outcomes implementing an electronic pharmacy care coordination queue for treatment of hepatitis C virus

**DOI:** 10.1093/ofid/ofaf695.2012

**Published:** 2026-01-11

**Authors:** Sheila Montalvo, Elizabeth Sherman, Paula A Eckardt, Eka Beriashvili, Alecia Muwonge, Jianli Niu, Kenneth K Poon, Garrett Van Ostran, Romina J Bromberg, Angela Savage, Elizabeth Thibodeau, Edison Cano Cevallos

**Affiliations:** Memorial Hospital System, Cooper City, FL; Memorial Healthcare Systems, Hollywood, Florida; Memorial Healthcare System, Hollywood, FL; Memorial Healthcare System, Hollywood, FL; Memorial Healthcare System, Hollywood, FL; Memorial Healthcare System, Hollywood, FL; Memorial Healthcare System, Hollywood, FL; Memorial Healthcare Systems, Hollywood, Florida; Memorial Healthcare System, Hollywood, FL; Memorial Healthcare Systems, Hollywood, Florida; Memorial Healthcare System, Hollywood, FL; Memorial Healthcare System, Hollywood, FL

## Abstract

**Background:**

To date, only 37% of Americans have been cured of chronic hepatitis C virus (HCV). Yet, the CDC’s strategic plan for HCV elimination includes the goal of viral clearance for 80% of people - a goal often stymied by structural barriers such as medical provider capacity and insurance restrictions. We hypothesized implementation of a pharmacy care coordination queue (PCCQ) would reduce barriers to HCV treatment success via co-management between medicine and pharmacy disciplines in a hospital-based infectious disease outpatient program.

**Figure 1.**
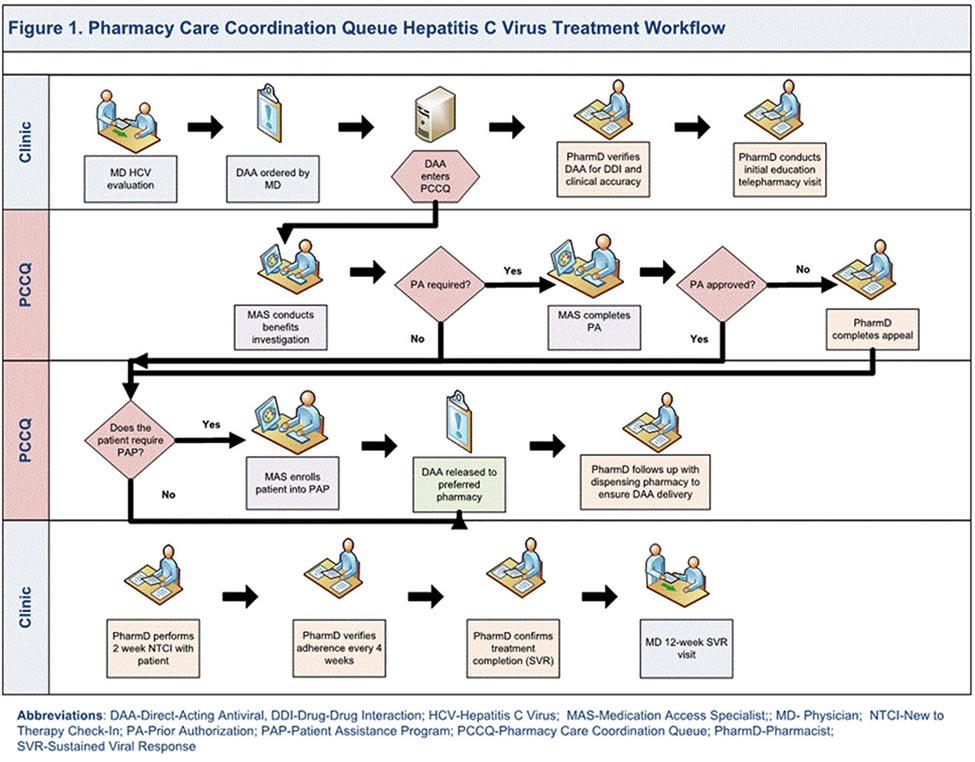
HCV PCCQ Workflow

**Tablet 1.**
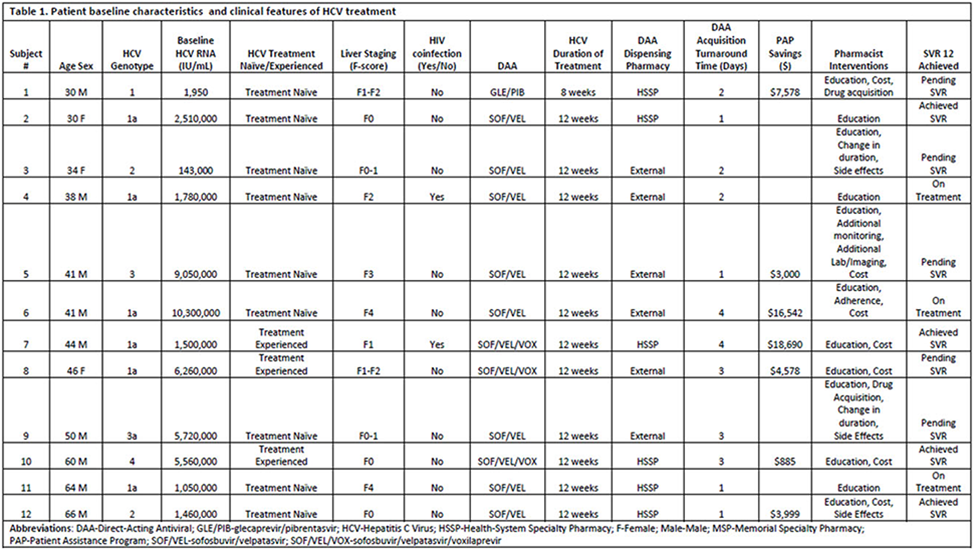
Patient baseline characteristics and HCV treatment outcomes

**Methods:**

People with chronic HCV were evaluated and prescribed direct acting antivirals (DAAs) by ID physicians and referred for co-management with the hospital-based specialty pharmacy program. The PCCQ (Figure 1) utilized pharmacy technicians and ID/HIV-trained clinical pharmacists. Pharmacy technicians managed tasks related to DAA acquisition including prior authorizations, appeals, and patient assistance programs (PAPs). Clinical pharmacists provided initial patient education, on-treatment follow-up of DAA tolerability, DAA and lab adherence, adverse reaction management, and facilitated engagement with the care team.

**Results:**

During the first 6 months of program implementation (Oct 2024-Apr 2025), 12 patients were co-managed through the PCCQ (Table 1). Patients were 75% male with a mean age of 45 (SD = 12) years. Genotype 1 HCV was most common (58%), followed by genotype 2 (17%) and genotype 3 (17%). HIV co-infection was present in 17% of patients, 25% of patients had F3-F4 liver fibrosis and 25% of patients were DAA treatment-experienced. DAA regimens prescribed were SOF/VEL (67%), SOF/VEL/VOX (25%), and GLE/PIB (8%). Turnaround time from writing the DAA prescription to DAA dispensing was mean 3 (SD = 1) days. The PCCQ obtained $55,272 in PAPs. Pharmacists made interventions on all 12 patients (Table 1). Of 12 patients, 4 (33%) completed treatment and achieved SVR, 5 (42%) completed treatment and are pending SVR, and 3 (25%) are still receiving treatment.

**Conclusion:**

Implementation of a PCCQ can expand medical provider capacity and positively affect patient access to and shorten time to DAA initiation, facilitate engagement in care and successful completion of therapy, and mitigate structural barriers and gaps in the HCV care cascade.

**Disclosures:**

All Authors: No reported disclosures

